# Self-Gathering Effect of the Hydrogen Diffusion in Welding Induced by the Solid-State Phase Transformation

**DOI:** 10.3390/ma12182897

**Published:** 2019-09-07

**Authors:** Zhiliang Xiong, Wenjian Zheng, Liping Tang, Jianguo Yang

**Affiliations:** 1 State Key Laboratory of Nuclear Power Safety Monitoring Technology and Equipment, China Nuclear Power Engineering Co., Ltd., Shenzhen, Guangdong 518172, China (Z.X.) (L.T.); 2 China Nuclear Power Design Co., Ltd. (Shenzhen), Shenzhen, Guangdong 518172, China; 3 Institute of Process Equipment and Control Engineering, Zhejiang University of Technology, Hangzhou 310014, China

**Keywords:** hydrogen diffusion, self-gathering effect, finite element method, welded joint, phase transformation

## Abstract

The hydrogen diffusion in welding was investigated by using thermal-mechanical-hydrogen diffusion sequential coupled procedures based on finite element method. A self-gathering effect induced by the solid-state phase transformation was discovered. Because of the self-gathering effect, the hydrogen concentration in weld metal was accumulated to a peak value which can be larger than the initial hydrogen concentration in molten pool, and subsequently the hydrogen concentration in heat affect zone was redistributed. In multi-pass welding, the gathered effect not only happened inside a weld pass, but also in the inter-pass, which further increased the sensitivity of the hydrogen-assisted cold cracking. Controlling should be adopted to restrain the hydrogen accumulation. Welding stress evolution during the solid-state phase transformation process had limited effect on the hydrogen diffusion.

## 1. Introduction

High strength steel (HSS) usually requires manufacture processes and assembling methods in fabrication industry, such as welding, coating, additive manufacturing, etc. The hydrogen-assisted cold cracking (HACC) is one of the most common defects, because the uptake of hydrogen from outside is inevitable. Because of the complexity of the welding and hydrogen atoms movement, the investigation of hydrogen diffusion in the welded joint faces a great challenge [1,2,3,4]. Nowadays, the precise mechanism of hydrogen-induced cracking is still non-unified, because there are kinds of models about material degradation in hydrogen with their respective supporting experimental results [5,6,7,8,9]. However, it is generally agreed that the occurrence of hydrogen-induced cracking requires several factors. For the HACC in welded joint, the factors are hardened microstructures such as martensite and bainite, residual stress and the hydrogen introduction from the atmosphere [10]. Inhomogeneity of the three conditions makes the hydrogen diffusion to be more complex. Up to now, there has not been an effective in situ testing method to detect the hydrogen concentration inside the welded joint. Most of the studies were concentrated on the hydrogen effect on the welded joint after the welding [11,12,13,14,15,16,17,18,19], rather than during the welding.

The solid-state phase transformation (SSPT) has significant influences on the residual stress in the welding [20,21,22,23]. The volume change induced by the SSPT greatly affects the stress state, especially in the cooling process, the expansion induced by the undercooled austenite to martensite phase transformation could even change the tensile stress into compressive stress in the welded joint. Similarly, the SSPT must affect the hydrogen diffusion in welding, because hydrogen diffusion property differences also exist in different phases and hydrogen diffusion can be driven by stress. But so far, there has not been any report about the precise effects of the SSPT on the hydrogen diffusion in welding. Researches have been conducted by numerical methods on the hydrogen diffusion in the welded joint [17,24,25], mostly focused on the effects of temperature field, few analyses took into account the interaction of transient stress field, phase transformation, and hydrogen diffusion. In addition, during these numerical approaches, same material properties were used in both heating and cooling processes, indicating that the influence of SSPT was neglected. The hydrogen diffusivity in ferritic steels (~10^−9^ m^2^/s in pure α iron [26], 10^−10^–10^−12^ m^2^/s in low alloy steels [27]) is several orders of magnitude larger than that in austenitic steels (1.8–8.0 × 10^−16^ m^2^/s in [28,29]), while the solubility of the austenitic steels (4–10 ppm MPa^−1/2^ [29,30]) is hundreds or even thousands of times larger than that in ferritic steels (~0.033 ppm MPa^−1/2^ in low alloy steels [31,32,33]). Therefore, the hydrogen diffusion during the SSPT is fairly different with that without considering the SSPT effects. Because of the importance of the hydrogen diffusion, its mechanism considering the SSPT effects, and subsequent influences on the welded joint should be carefully investigated. 

As mentioned, the hydrogen diffusion inside the welded joint cannot be in situ measured by the experiment methods, including the traditional test methods [34,35,36,37] and advanced in situ observation technology [38,39,40]. The finite element analysis is probably the only effective method to predict the hydrogen concentration evolution. Although few studies have been reported in this field [17,24,25], the SSPT effects have never been considered. In this study, numerical approaches were conducted to investigate the hydrogen diffusion in both single-pass and multi-pass welding. The effects of SSPT on the thermal field, stress field, and especially hydrogen concentration field were obtained. A self-gathering effect was discovered in the welded joint where the hydrogen diffused uphill to a value which might be much larger than the initial hydrogen concentration in the molten pool. The reason and influence factors for this effect were investigated in both.

## 2. Investigation Procedure

### 2.1. Modelling

**Figure 1:**
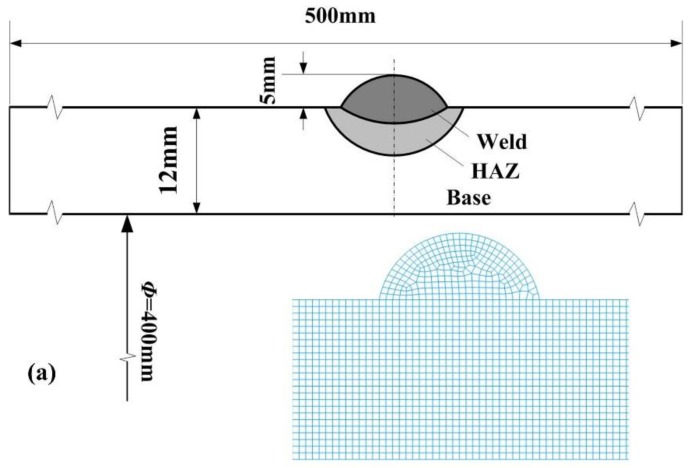

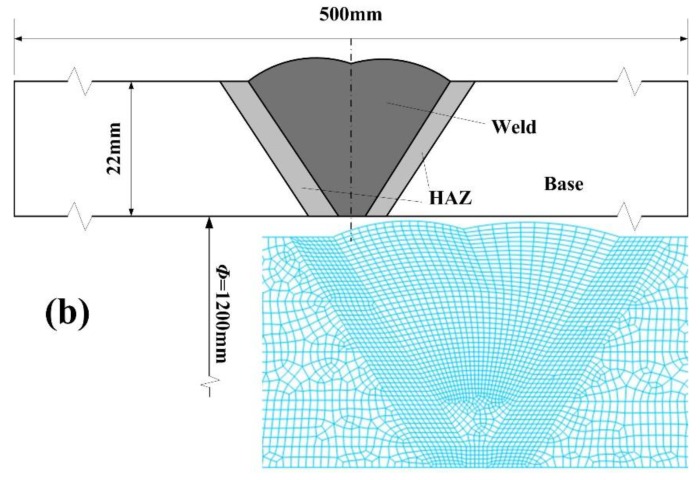
Sketch of the welded joints and mesh models (**a**) single-pass; (**b**) multi-pass.

In this study, a X80 pipeline steel was used as the base material, a single-pass surfacing and a multi-pass welding were carried out by using conventional gas metal arc welding (GMAW). The dimensions of the weldment and welded joint are shown in Figure 1.

**Figure 2:**
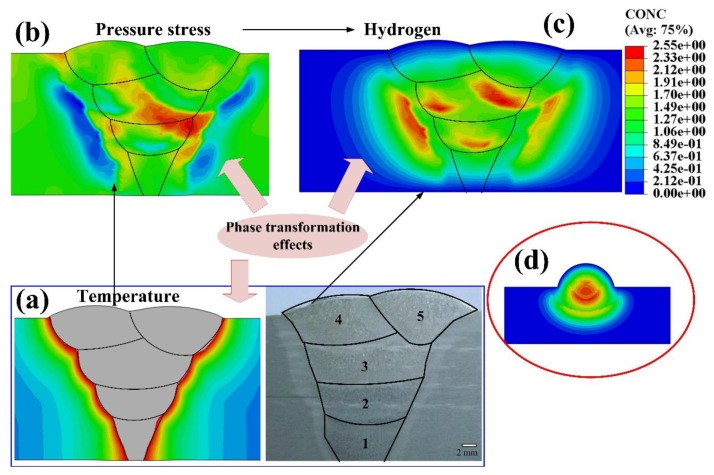
Hydrogen diffusion simulation procedure (**a**) peak temperature and weld pass distribution [38]; (**b**) pressure stress distribution; (**c**) hydrogen concentration distribution in multi-pass; (**d**) hydrogen concentration distribution in single-pass.

Thermal-mechanical-hydrogen diffusion sequential procedures were built to investigate the hydrogen diffusion during the whole welding process, as shown in Figure 2. First, a heat transfer procedure was built up to investigate the temperature distribution in the welding, temperature evolutions were saved. In this analysis, a volumetric heat source which has been adjusted by experimental results [41] was used as the welding resource, as seen in Figure 2a. Radiation and convection on the outside surface were used to consider the heat loss in welding. Second, the stress and strain evolutions were calculated by elastic-plastic mechanical analysis which used the Von Mises yield criterion and associated hardening rule. The temperature history obtained by the previous step was added into this analysis as a predefined field. The volume reduction in heating process and expansion in cooling process induced by the SSPT were also considered. The pressure stress evolutions were saved, as shown in Figure 2b. Lastly, the temperature and pressure stress evolutions were input as predefined field into mass diffusion simulation to obtain the hydrogen transient distribution in welding, as shown in Figure 2c. In this study, single-pass welding (Figure 2d) was used to investigate the hydrogen diffusion mechanism inside of a single welded joint, multi-pass welding was used to study the hydrogen diffusion in different weld passes. All the simulations used axisymmetric models and were performed by the ABAQUS/Explicit 6.13 with Fortran subroutines.

### 2.2. Material Properties Considering the Effect of SSPT

As known, the microstructure of X80 pipeline steel usually consists of ferrite phases, such as ferrite, pearlite, bainite, martensite, etc. These phases have body-centered cubic (bcc) or body-centered tetragonal (bct) structure. During the heating process of the welding, the ferrite phases undergo a phase transformation to austenite phase which has face-centered cubic (fcc) structure at temperature above *A_c_*_1_ (cementite disappearance temperature). While in the cooling process, the fcc structure austenite phase changes back to ferrite phase at a relative low temperature related to the cooling rate, chemical component of the material, etc. To describe this phase state evolution during the welding, a user-defined field variable was used in simulation. This field variable was defined by the relationship of the phase transformation temperature and the welding heat cycle curve, namely ferrites, austenite, or mixture. By using this field variable, the phase transformation evolution of the welded joint during the whole welding process can be obtained. 

The material properties were set as phase dependent. The phase state was judged first by the phase field variable, and then the corresponding material properties were invoked. The thermal and mechanical material properties with ferrite phases and (undercooled) austenite phase are shown in Table 1 and Table 2, respectively. As seen, the material properties were also temperature dependent.

**Figure 3:**
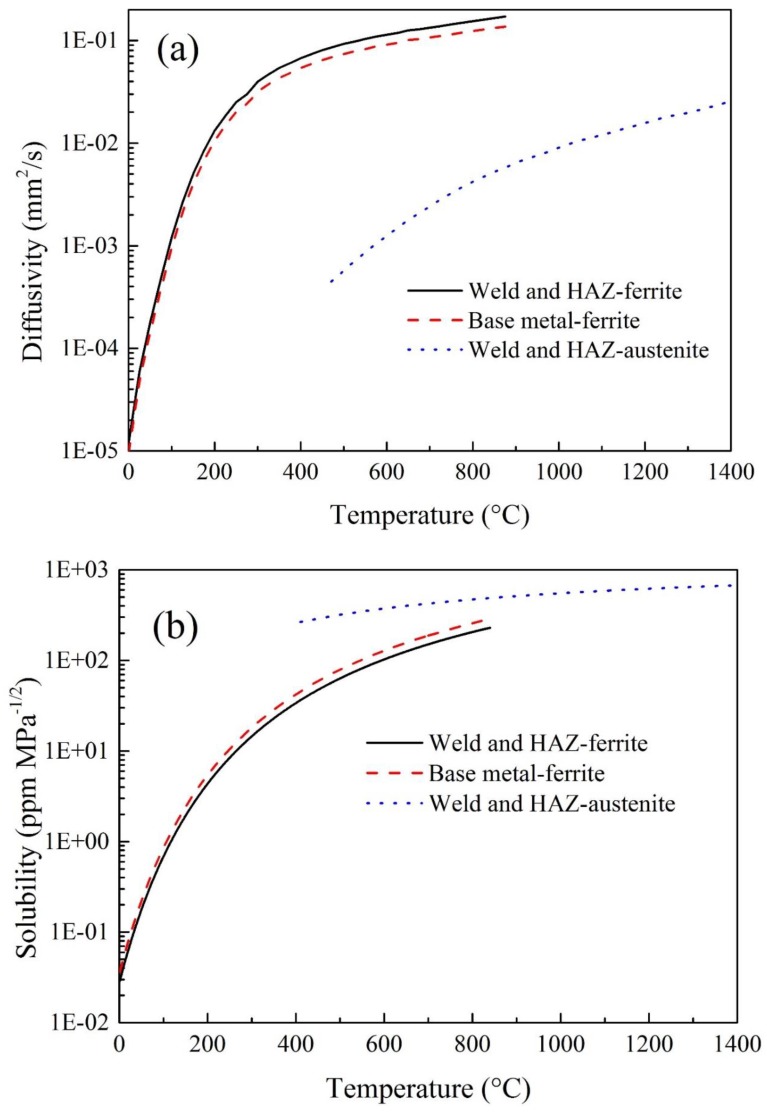
(**a**) Hydrogen diffusivity and (**b**) solubility.

The welded joint consists of weld metal (WM), heat affected zone (HAZ), and base metal (BM). The chemical constituents of the weld metal are similar to that of the base material, thus the thermal and mechanical properties of weld and HAZ were assumed to be the same as that of the base material. However, for the hydrogen diffusion properties, the differences cannot be neglected. The BM manufactured by TMCP process contains lots of anti-diffusion defects, shows a lower diffusivity and a higher solubility. While the WM has a larger diffusivity and lower solubility because of the as-cast microstructures have less density of anti-diffusion defects. The HAZ underwent the high temperature heat treatment. Its diffusivity is similar to or slightly less than that of the BM, which has been verified in experimental results [16,42]. For simplification, the hydrogen diffusion properties of the HAZ were assumed to be the same as that of the WM. The hydrogen diffusivity and solubility of different phases at different zones of the welded joint are shown in Figure 3. For the lack of the hydrogen diffusion properties at elevated temperature, the difference in hydrogen diffusion properties between the welded and base metals at elevated temperature was assumed to be the same as the difference at room temperature. Here, the hydrogen diffusivity of the weld metal/HAZ was set 1.25 times that of the base metal, while the solubility of the base metal was also set 1.25 times that of the weld metal/HAZ. Note that it is hard to obtain the hydrogen diffusion properties cover the whole temperature range from ambient temperature to molten liquid temperature, experimental results from literature [12,17,18,25,41,42] and extrapolation method were used to define the hydrogen diffusion properties. Because of the large differences of hydrogen diffusion property in different phases, the defined properties showed in Figure 3 might be less precise, but enough to reveal the influences of SSPT on hydrogen diffusion in welding.

Before or after the SSPT process, the material properties can be directly defined by using the field variable. However, during the SSPT processes, both in heating and cooling, the mixture material contains two kinds of phases, i.e., ferrite phase and austenite or undercooled austenite phase. One effective thermal-mechanical-hydrogen diffusion property of the mixture material *P_eff_* was defined to be associated with the phase fraction of austenite and ferrite phases in Equations (1) and (2),

(1) $$P_{eff}=f_{\gamma }P_{\gamma }+f_{\alpha }P_{\alpha }$$

(2) $$f_{\gamma }+f_{\alpha }=1$$

where *f_α_* and *f_γ_* are the phase fraction of the ferrite phases and austenite phase, respectively. *P_α_* and *P_γ_* are the property of the ferrite phases material and austenite phase material at the corresponding temperature, respectively. In heating stage, the phase fraction was reflected by a simplified linear relationship with superheat temperature. While in cooling stage, the phase fraction was set as a function of temperature obtained by the experiment. Because the range of cooling rate is very narrow in this welding, because of which a preheat treatment of 150 °C was used, the limited effect of the cooling rate can be neglected. Thus, by using the above definition of material properties, the effects of SSPT can be considered in the simulation procedures.

Besides, the volume changes induced by the phase transformations were also considered in the mechanical analyses, the shrinkage and expansion strains in heating and cooling process are −2.3 × 10^−3^ and 7.4 × 10^−3^, respectively. For simplicity, linear approximation was used to describe the strain increment with temperature increment during the phase transformation processes [20,21].

### 2.3. Hydrogen Diffusion Analysis

The driving forces of hydrogen diffusion are normalized concentration *ϕ* which equals to the ratio of hydrogen concentration to solubility *c*/*s*, temperature gradient and pressure stress gradient, as shown in the governing Equation (3) [43,44,45]:

(3) $$J=-sD\left\{\nabla \phi +\kappa_{s}\nabla \left[\ln(T-T_{0})\right]+\kappa_{p}\nabla p\right\}$$

where *J* is the flux of concentration of hydrogen, *D* and *s* are the hydrogen diffusivity and solubility, respectively. *T* is the temperature in degrees centigrade, and *T*_0_ is the absolute zero. The Soret effect factor, *κ_s_*, providing diffusion because of temperature gradient, is given by Equation (4):

(4) $$\kappa_{s}=\frac{c(T-T_{0})}{s^{2}}\frac{\partial s}{\partial T}$$

The pressure stress factor, *κ_p_*, providing diffusion driven by the gradient of the equivalent stress *p*, is shown as Equation (5):

(5) $$\kappa_{p}=\frac{\bar{V_{H}}\phi }{R(T-T_{0})}\;\mathrm{mm}\;N^{-1/2}$$

where *R* is the universal gas constant, $\bar{V_{H}}$ = 2.0 × 10^3^ mm^3^/mol is the partial molar volume of the hydrogen in iron-base metals [46]. The initial hydrogen concentration in the molten pool was set as 5 ppm, and 0 ppm on the outer surface.

## 3. Results and Discussions

### 3.1. Single-Pass Welding

**Figure 4:**
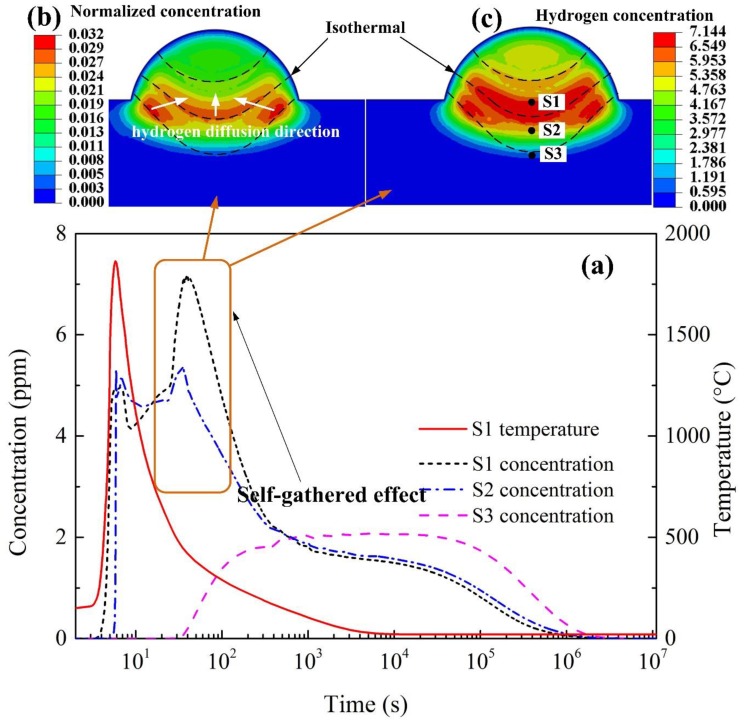
Self-gathering effect in single-pass welding; (**a**) hydrogen concentration evolution of critical positions during the whole welding process; (**b**) normalized concentration distribution during the SSPT; (**c**) hydrogen concentration distribution during the SSPT.

Figure 4 shows the hydrogen diffusion evolution of the single-pass welding. Figure 4a gives the hydrogen concentration over time at different points in the single-pass welded joint. Figure 4b,c shows the normalized concentration distribution and hydrogen concentration distribution during the SSPT, respectively. Critical positions are labeled in Figure 4c, S1—center point of weld metal, S2—point at fusion line, and S3—point at HAZ. In cooling process, the hydrogen in the weld metal diffused outside or to the hydrogen-free material. In previous studies without considering the effect of SSPT [17,24,25], the hydrogen concentration in the whole WM decreased continuously after the solidification of the molten pool. However, in our investigation, with the influence of SSPT, the hydrogen concentration of the center domain of the WM increased in the temperature range of SSPT. At some critical position, such as S1, the maximum of the hydrogen concentration can reach a value even larger than the initial hydrogen concentration of the molten pool. It seems that the WM redistributed hydrogen concentration by itself during SSPT process, without the outside intervention. Here, this phenomenon was named self-gathering effect, as shown in the box of Figure 4a. This self-gathering effect directly caused uphill diffusion for the hydrogen concentration at critical positions of the WM.

In the cooling process, the isothermal moved along the temperature gradient direction, as well as the sequence of decomposition of the undercooled austenite. The SSPT happened from HAZ to WM with sharply decreasing the solubility and increasing the diffusivity. Especially in the WM, the normalized concentration in the transformed ferrite phase increased obviously because of the sharply decreased solubility; even its hydrogen concentration was lower, while the normalized concentration in the undercooled austenite was basically unchanged. Thus, through the SSPT interface, the hydrogen diffused from the pre-decomposed ferrite phases side to the undecomposed undercooled austenite phase side, causing an increase in the hydrogen concentration in the latter phase, i.e., uphill diffusion as shown in Figure 2a. Therefore, the self-gathering effect was caused by the sharp change in the hydrogen diffusion properties during SSPT, the residual stress had less effect which is discussed in detail in Section 3.3. After the SSPT, because of the difference of solubility (normalized concentration) induced by the temperature gradient, the uphill diffusion might still happen with a higher diffusivity in ferrite phases, until the maximum of concentration is reached. Hence, the self-gathering effect is mainly attributed to the change of hydrogen diffusion properties induced by the SSPT.

### 3.2. Multi-Pass Welding

**Figure 5:**
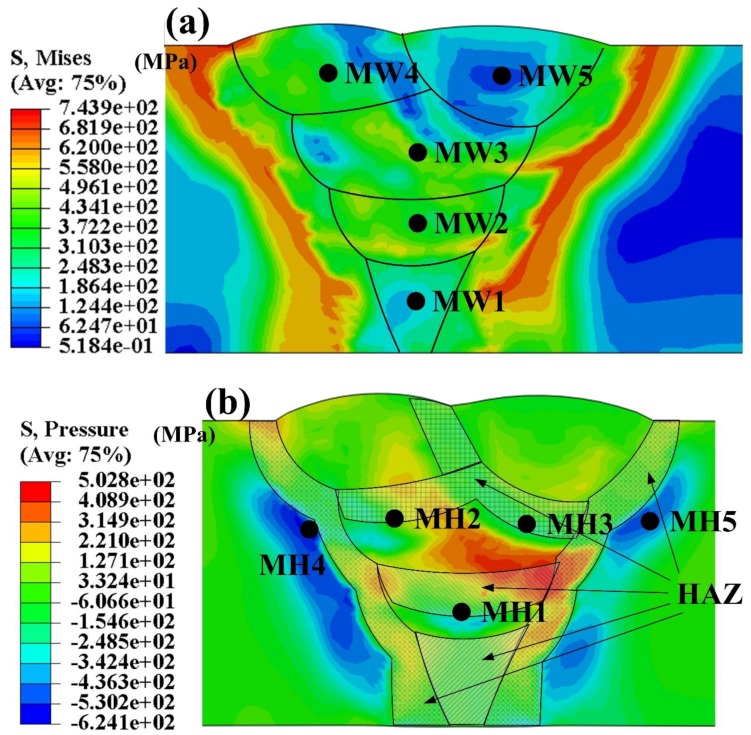
Residual stress distribution of the multi-pass welded joint; (**a**) Mises; (**b**) pressure stress.

Figure 5 shows the residual stress distribution of the multi-pass welded joint. The phase transformation from austenite to ferrites makes volume expansion of the material by opposing the thermal contraction in the cooling process. According to the thermal simulation experiments, the expansion strain change of SSPT in cooling is limited in the cooling rate range of 10–30 °C/s which covers the cooling rate range in this welding. Thus, the expansion strain was set as a constant for convenience. Because of this expansion, the residual stress level of the WM decreased, as the Mises stress results shown in Figure 5a. The stress level in the WM is obviously lower than that in HAZ, especially in the last two weld passes. The longitudinal stress in the WM, as shown in Figure 5b, was even changed from tensile stress to compressive stress [23]. The stress concentration area was in the HAZ of the whole welded joint, exactly in the inter-critical HAZ (ICHAZ). In addition, the tensile stress also existed in the inter-pass HAZs, such as the labeled positions in Figure 5b.

**Figure 6:**
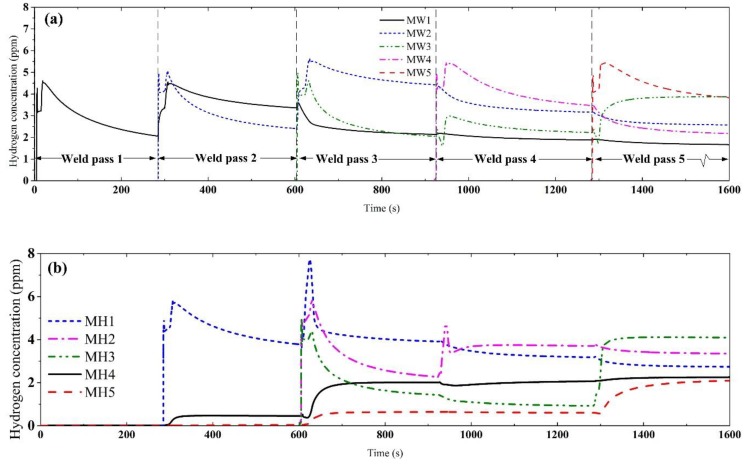
Hydrogen diffusion evolution of the multi-pass welded joint during the whole welding process. (**a**) Positions in WM as labeled in Figure 5a; (**b**) positions in HAZ as labeled in Figure 5b.

Figure 6 shows the hydrogen diffusion evolution in the multi-pass welding. The hydrogen diffusion evolution at the positions inside of the weld passed (labeled in Figure 5a) are shown in Figure 6a. The welding process of each single-pass is marked. Hydrogen concentration of one weld pass can be influenced by several following welding processes. Thus, the hydrogen concentration in the WM of weld pass can strongly fluctuate as the welding time. Obviously, there was self-gathering effect in each weld pass which is similar to that in single-pass welding. However, the hydrogen concentration evolution in each weld pass is different. To be precise, the maximum of the hydrogen concentration in subsequent weld pass was larger than that in former weld pass, and the decreasing rate is slower than that in single-pass welding. This difference was caused by the self-gathering effect in inter-pass. In the welding process of a new weld pass, the microstructure in new weld pass was austenite, while ferrite in the former weld passes, the retained hydrogen in former weld passes caused much higher normalized concentration compared to that in the new weld pass. Thus, the hydrogen was transferred from former weld pass/passes to the new one, either in heating or cooling process. 

The hydrogen concentration evolutions in HAZ and inter-pass HAZ are shown in Figure 6b; the positions are labeled in Figure 5b. The hydrogen concentration in the inter-pass HAZ was mainly influenced by the gathered effect in inter-pass, and the peak value was larger than that inside the weld pass during the SSPT.

**Figure 7:**
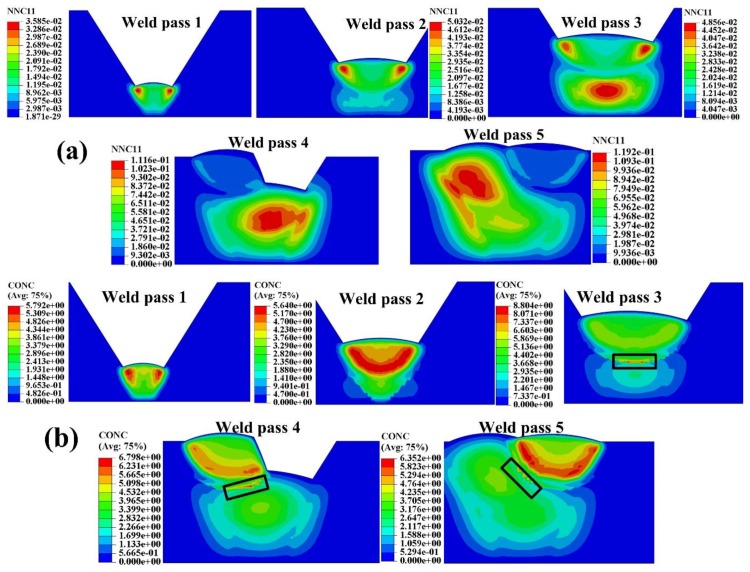
Characteristics of the Self-gathering effect in different weld pass welding (**a**) normalized concentration during the SSPT; (**b**) hydrogen concentration during the SSPT.

Figure 7 gives the characteristics of the self-gathering effect in different weld pass welding. In the welding of the first two weld passes, the self-gathering effect inside the weld was dominating. As the weld pass number increased, self-gathering effect in inner-pass became obvious. Because of the self-gathering effect in inner-pass, the hydrogen was transferred toward latter pass for the hydrogen diffusivity in ferrites is much larger than that in austenite. The hydrogen was accumulated in the austenite side next to the ferrites, as shown in the black wireframe in Figure 7b.

The gathered hydrogen concentration highly affects the welded joint. First, the higher hydrogen concentration might cause degradation of the material even at elevated temperature, especially for the quenched microstructures which had been formed at this movement. Second, HAZ is generally the weakest position of the welded joint with a higher occurrence probability of the HACC, especially for the high-strength steels. The gathered hydrogen concentration in WM directly increased the hydrogen concentration level of the HAZ in the subsequent diffusion process, afterward increased the susceptibility of HACC. In multi-pass welded joint, because of the self-gathering effect inside the weld pass and in inter-pass, the hydrogen concentration in the inter-pass HAZ was much higher, causing higher susceptibility of HACC. In addition, the subsequent weld pass always gathered the hydrogen from the former passes. With increasing the weld pass number in thick plate welding, the hydrogen concentration in the last several weld passes should be the highest in the whole welded joint. As shown in Figure 6a,b, the retained hydrogen concentration in the 5th welded joint was the highest during the whole welding process of this multi-pass joint. That agrees with the experimental results where the HACC were initiated at a distance away from the top of the WM surface in multi-pass welded joint [47], indicating that the self-gathering effect partially contributes to this phenomenon. In a previous study [25], the hydrogen distribution in the welded joint of thick plate was investigated by experimental methods and numerical methods without considering the SSPT effect. Although the hydrogen distribution along the thickness obtained by the two methods was similar in shape, the average hydrogen amount in local area lost the features of the peak value with its position and the gathered degree. 

### 3.3. Influence of the Welding Stress

It can be judged that the self-gathering effect is harmful to the welded joint. Decreasing the hydrogen gathered degree is very significant, especially for high or ultra-high strength steels. In welding, it generally takes less than 10s for the SSPT process; however, in this time range, the maximum diffusion distance is less than 1 mm. Thus, the SSPT process is faster than the hydrogen diffusion. Hydrogen gathered degree is influenced by the material properties, welding process, heat treatment, phase transformation kinds, and temperature range, etc. Control measures should be further investigated to decrease the accumulation of the hydrogen concentration, such as decreasing the decomposition temperature of the undercooled austenite, narrowing the temperature range of SSPT in cooling process, reducing the difference of the material properties before and after the SSPT, and so on. Among them, controlling the welding residual stress is believed to be useful in the traditional mindset because of the diffusion driven by stress gradient. Furthermore, if the welded joint was under hydrogen environment, the hydrogen concentration in WM could be up to several times higher than that without influence of residual stress [42].

**Figure 8:**
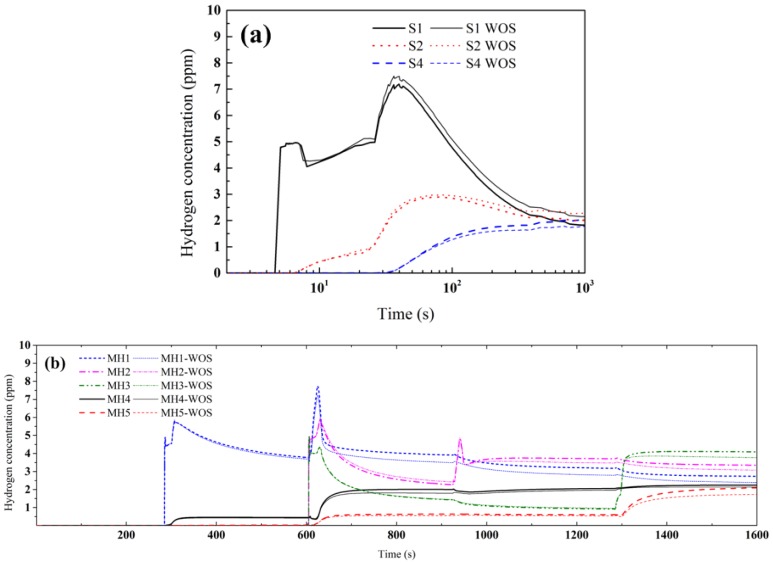
The comparison of hydrogen diffusion evolutions with and without considering the stress (WOS) in single-pass (**a**) and multi-pass welding (**b**).

To reveal the influence of the welding stress on self-gathering effect, the hydrogen diffusion simulations of single-pass and multi-pass welding were conducted again just considering the temperature evolution. Thus, the obtained hydrogen diffusion evolutions did not consider the influence of welding stress. The comparison of hydrogen diffusion evolutions is shown in Figure 8. The hydrogen concentration evolution without considering the welding stress effect was consistent with that considering the welding stress effect. The uphill hydrogen diffusion still existed, hydrogen concentration value just slightly changed, both in single-pass and multi-pass welding.

During the SSPT process, the strength of the material declined greatly and caused low level stress in the transformation temperature range, and more importantly, the pressure stress factor (seen Equation (5)) is small enough because of the large temperature *T* and small value of normalized concentration *ϕ*. Both the reasons limited the effect of welding stress on the hydrogen diffusion. Therefore, the self-gathering effect dominated the hydrogen diffusion, while the influence of the welding stress was limited. 

Some kinds of treatments during welding redistribute the residual stress, such as trailing impact and rolling. It has been proven that these treatments can prevent HACC of HSS. However, according to our results, the hydrogen concentration distribution cannot be changed obviously before the welded joint cools down, even by the redistributing welding stress. The temperature of the welded joint was relatively high during SSPT. The effect of the stress-driven diffusion was limited for the pressure stress factor is very small at elevated temperatures. Although the redistributed residual stress and plastic strain caused by the stress redistribution treatments have positive effects on the prevention of HACC or delays hydrogen embrittlement damage [48,49,50], those effects would not apparently present before the welded joint cools down. Thus, the accumulated hydrogen concentration induced by the self-gathering effect is hardly changed by the stress redistribution treatments at elevated temperatures, and other effective controlling should be further studied.

## 4. Conclusions

The hydrogen diffusion in the single-pass and multi-pass welding was predicted by a thermal-mechanical-hydrogen diffusion sequential coupled procedure considering the SSPT effects in a high strength X80 pipe steel. An obvious uphill hydrogen diffusion induced by the self-gathering effect was found in both kinds of welded joints. The self-gathering effect was mainly caused by the great change of the hydrogen diffusion properties during the SSPT process. This effect not only happens inside the weld pass, but also in the inter-pass. Thus, there exists a trend that the latter weld pass always gathers hydrogen from the former pass or passes in multi-pass welding. For the phenomenon that the most sensitive position of HACC is located at a distance away from the top of the WM surface, the self-gathering effect induced by the SSPT might provide partial contribution. The welding stress has limited effect on the hydrogen diffusion during the SSPT process. To decrease the sensitivity of HACC in welded joint, controlling should be used to weaken the accumulated level of the hydrogen.

In engineering, a critical hydrogen concentration was artificially defined to prevent the decrease of strength or emergence of hydrogen-induced cracking for HSS or welded joint. However, the measured results are the average value in a bulk of material. The self-gathering effect causes heterogeneous distribution of hydrogen. Thus, both the mean value and the peak value should be considered in designing controlling. In addition, reducing the self-gathering effect is necessary, its influence factors and the effective measures should be further investigated. 

## Figures and Tables

**Table 1:** Thermal and mechanical material properties of ferrite phases.

Material Properties	Temperature (°C)						
	20	100	200	400	600	700	850
Conductivity (W/m·°C)	54.4	54.0	52.8	47.7	40.0	33.4	27.3
Specific heat (J/kg·°C)	423	473	478	492	530	650	682
Young’s modulus (GPa)	215	207	204	185	166	134	118
Harden modulus (GPa)	1.4	1.3	1.3	1.2	1.1	1.1	1.0
Yield stress (MPa)	641	623	597	550	451	275	98
Thermal expansion coefficient (1 × 10^−5^)	1.10	1.15	1.22	1.37	1.42	1.46	1.49
Density (g/cm^3^)	7.81	7.79	7.77	7.72	7.69	7.66	7.61
Possion’s ratio	0.29	0.30	0.30	0.30	0.31	0.32	0.32

**Table 2:** Thermal and mechanical material properties of austenite or undercooled austenite phase.

Material Properties	Temperature (°C)						
	400	600	700	800	900	1200	1400
Conductivity (W/m·°C)	18.0	19.2	22.5	24.0	28.1	32.2	34.0
Specific heat (J/kg·°C)	540	570	584	608	632	676	700
Young’s modulus (GPa)	167	163	156	145	132	60	10
Harden modulus (GPa)	1.4	1.3	1.1	0.9	0.8	0.5	0.3
Yield stress (MPa)	160	141	123	101	60	25	10
Thermal expansion coefficient (1 × 10^−5^)	1.8	1.9	2.0	2.0	2.0	2.1	2.2
Density (g/cm^3^)	7.75	7.66	7.61	7.56	7.46	7.37	7.32
Possion’s ratio	0.32	0.32	0.33	0.33	0.33	0.34	0.39
